# Where to Forage in the Absence of Sea Ice? Bathymetry As a Key Factor for an Arctic Seabird

**DOI:** 10.1371/journal.pone.0157764

**Published:** 2016-07-20

**Authors:** Françoise Amélineau, David Grémillet, Delphine Bonnet, Tangi Le Bot, Jérôme Fort

**Affiliations:** 1 CEFE UMR 5175, CNRS – Université de Montpellier – Université Paul-Valéry Montpellier – EPHE, Montpellier, France; 2 Percy FitzPatrick Institute, DST/NRF Centre of Excellence, University of Cape Town, Rondebosch, South Africa; 3 Laboratoire MARBEC, Université de Montpellier, Montpellier, France; 4 Department of Bioscience, Aarhus University, Roskilde, Denmark; 5 Littoral Environnement et Sociétés (LIENSs), UMR 7266 CNRS-Université de La Rochelle, La Rochelle, France; Institute of Ecology, GERMANY

## Abstract

The earth is warming at an alarming rate, especially in the Arctic, where a marked decline in sea ice cover may have far-ranging consequences for endemic species. Little auks, endemic Arctic seabirds, are key bioindicators as they forage in the marginal ice zone and feed preferentially on lipid-rich Arctic copepods and ice-associated amphipods sensitive to the consequences of global warming. We tested how little auks cope with an ice-free foraging environment during the breeding season. To this end, we took advantage of natural variation in sea ice concentration along the east coast of Greenland. We compared foraging and diving behaviour, chick diet and growth and adult body condition between two years, in the presence versus nearby absence of sea ice in the vicinity of their breeding site. Moreover, we sampled zooplankton at sea when sea ice was absent to evaluate prey location and little auk dietary preferences. Little auks foraged in the same areas both years, irrespective of sea ice presence/concentration, and targeted the shelf break and the continental shelf. We confirmed that breeding little auks showed a clear preference for larger copepod species to feed their chick, but caught smaller copepods and nearly no ice-associated amphipod when sea ice was absent. Nevertheless, these dietary changes had no impact on chick growth and adult body condition. Our findings demonstrate the importance of bathymetry for profitable little auk foraging, whatever the sea-ice conditions. Our investigations, along with recent studies, also confirm more flexibility than previously predicted for this key species in a warming Arctic.

## Introduction

The release of anthropogenic greenhouse gases into the atmosphere leads to climate warming on a worldwide scale [1]. Consequences are diverse among regions, yet the Arctic is arguably the most impacted area due to changes in the cryosphere. In particular, minimum summer sea ice extent decreased by 12 ± 2% per decade since 1979 [1]. Changes in the cryosphere significantly affect the Arctic biota [2]. For instance, observed and predicted declines in sea ice extent will affect animals that use sea ice as a habitat, such as seals, walruses (*Odobenus rosmarus*), polar bears (*Ursus maritimus*) or ice-associated amphipods [3,4]. Modifications in sea ice extent and in the timing of sea ice melting in spring will also perturb the amplitude, location and timing of Arctic plankton blooms [5]. These blooms are an essential feature of Arctic marine ecological processes, and the resulting stochasticity in primary and secondary productivity (phyto- and zooplankton biomasses) is predicted to impact higher trophic levels, including seabirds [6–8].

Little auks *(Alle alle)* are endemic to the Arctic and the most abundant seabird in the North Atlantic Arctic, with an estimated population of 40–80 million individuals [9]. Recent studies have demonstrated that they are affected by the ecological consequences of higher ocean temperatures in the Arctic [10–13]. Beyond ocean temperatures, little auks might also be affected by the presence/absence of sea ice. During the breeding season, this planktivorous species is known to use the marginal ice zone (the transition area between pack ice and open water), whenever accessible, to forage and to rest [14–18], a behaviour also suspected to occur outside of the breeding season [19]. Moreover, prey availability and species composition are predicted to differ significantly according to sea ice concentration (SIC, percentage of sea surface covered by ice in a given area), particularly in the case of ice-associated species [17]. Such organisms are the preferred prey of little auks, because of their high lipid concentration [20], and little auks feeding within Atlantic ice-free water masses have been found to forage in less optimal conditions due to smaller, leaner prey [11,12]. Yet marine productivity is also tightly linked to bathymetry [21]. In particular, continental shelves and shelf break slopes modify water fluxes and induce plankton concentration and aggregation of top predators [22,23]. Aggregations of little auks have been observed along the shelf-break outside the breeding season, probably reflecting an area of high prey density [24–26]. In the perspective of an ice-free Arctic Ocean in summer, bathymetry is, with light intensity, the environmental parameter that will remain unchanged. Understanding how little auks take advantage of bathymetric features is thus needed to predict climate change impacts on this species.

In this study, we tested the hypothesis that little auk foraging behaviour during the breeding season is affected both by the presence/absence of sea ice and bathymetry. To this end, we looked at the effects of these two factors on (1) little auk foraging location and diving activity, and (2) zooplankton species composition of chick diet. We also investigated their impact on (3) chick growth and adult body condition.

To test this hypothesis, we studied little auks from the breeding colony of Ukaleqarteq (Kap Höegh), located in East Greenland where foraging conditions are influenced by the East Greenland current carrying large volumes of Arctic sea ice southwards. Importantly, there is a strong inter-annual variability in this sea ice drift, allowing us to compare little auk foraging behaviour in the presence/nearly absence of sea ice within their foraging range (in 2012 and 2014, respectively). To this end, we used a multidisciplinary approach, combining satellite remote-sensing of sea ice concentration, land-based studies of little auk foraging behaviour and reproductive performance, and at-sea observations of little auk distribution and sampling of their zooplankton prey.

## Materials and Methods

The R software version 3.0.2 was used for numerical and statistical analyses [27]. QGis [28] was used to map GPS and sea-ice data. All bird handling procedures and at-sea samplings were approved by the Government of Greenland (Permits N°2012–065815 and 2014–098814) and validated by the ethics committee of the French Polar Institute (Permit N° MP/53/06/12).

### Field site and data collection

#### General fieldwork at the colony

**Fig 1:**
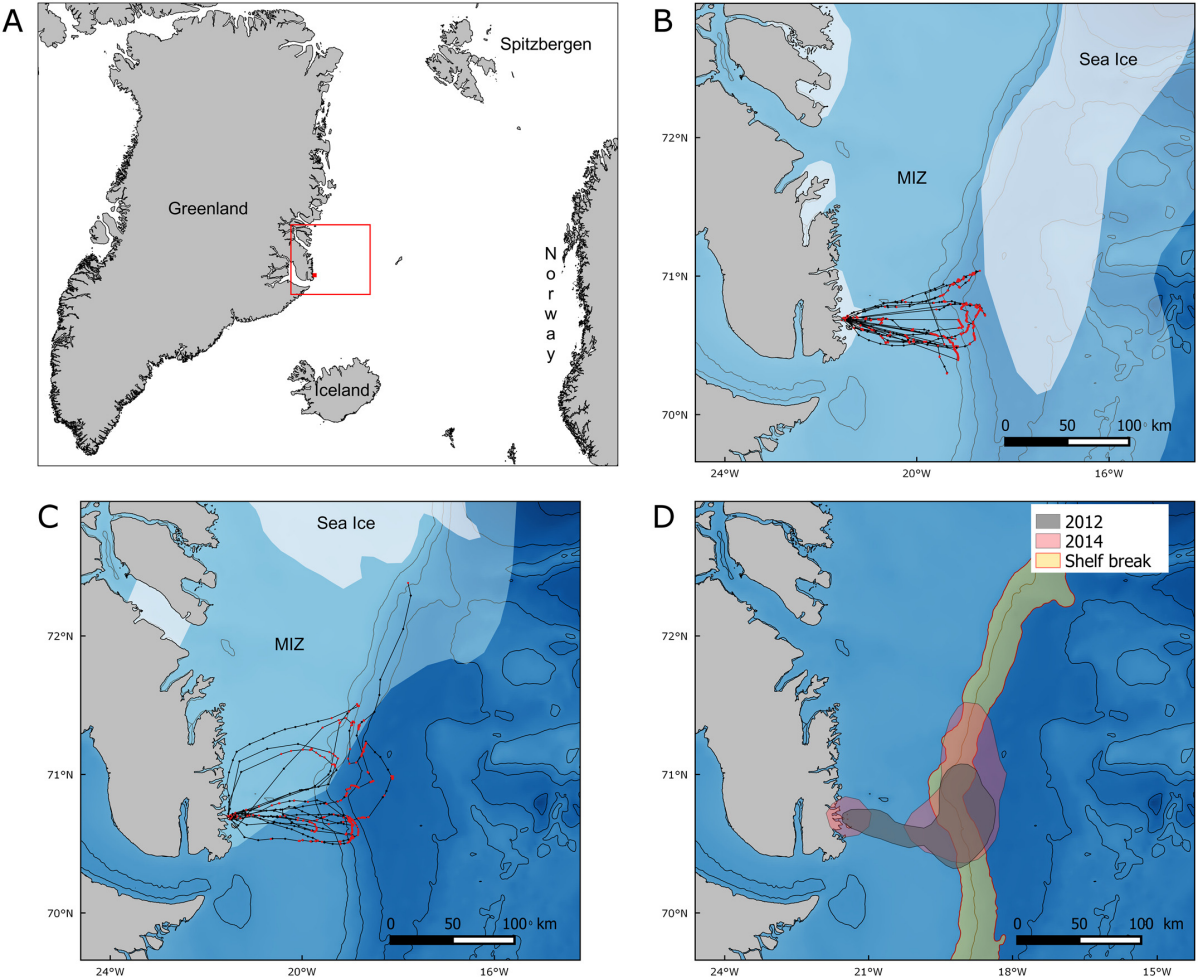
(A) Location of the study site, sea ice extent and GPS tracks from (B) 2012 and (C) 2014 and (D) 75% kernel contours of resting and foraging positions in 2012 (green), and 2014 (pink), and the shelf break area (yellow). 1A: General map situating Ukaleqarteq (red dot) and the location of the studied area (red rectangle) represented in Figs 1B, 1C, 5A and S1. 1B and 1C: GPS tracks: red dots correspond to foraging or resting (speed <10 km.h^-1^) and black dots to travelling (speed >10 km.h^-1^). Sea ice extent data were downloaded from the U.S. National Ice Center (http://www.natice.noaa.gov/products/daily_products.html, 24 July 2012 and 23 July 2014). White: pack ice with an ice concentration >80%. Light blue: marginal ice zone (MIZ) with an ice concentration <80%. In the marginal ice zone, sea ice concentration decreased between pack ice and open water. Black lines: 500-m isobaths. GPS track of the bird going far North-East in 2014 was not included in the analyses because it was not complete, but we present it on this map to show how this bird travelled along the shelf break and probably target areas of high ice concentration. 1D. Red lines represent the 500 and 1500m isobaths used to delimit the shelf break area. Projection: GR96/ UTM zone 27N.

Fieldwork took place in Ukaleqarteq (Kap Höegh, 70°44’ N, 21°35’ W, Fig 1A), East Greenland, between mid-July and mid-August 2012 and 2014.

Adult birds were caught either in the nest by hand or on rocks surrounding the nest using noose traps. Each handled adult bird was weighed (to the nearest gram), measured (head-bill and wing length to the nearest millimeter), fitted with a metal ring for individual identification and equipped with a data logger (see below; 2012: n = 38; 2014: n = 32). Additional breeding birds (2012: n = 27; 2014: n = 88) were captured and measured only following the same procedure to access their body condition. All birds were released within less than 10 minutes of capture. The breeding status of adult birds was ascertained via the presence of a full gular pouch (a sub-lingual pouch used for prey transport to the chick), of a brood patch, or through the presence of a chick at the nest. When birds had a full gular pouch, chick diet was collected following Harding et al [20] (2012: n = 20, 2014: n = 20). Little auks only raise one chick, and chick growth was monitored. To this end, some nests were visited every second day to determine hatching date (2012: n = 24, 2014: n = 29). Chicks were then weighted every second day, once they were more than 2 days-old.

#### Logger deployment

GPS-recorders or Temperature-Depth-Recorders (TDR) were attached dorsally (GPS) or ventrally (TDR) to feathers with Tesa^®^ tape (Hamburg, Germany). Devices were either removed upon recapture after 3–10 days, or fell off during the complete moult which immediately follows the breeding season in little auks [29]. Two GPS types were used: EP-3.3 in 2012 and 2014 (Ecotone, Gdansk, Poland; 40 x 17 x 9 mm, 4.9 g, 3.2% of the average little auk weight) and ALLE in 2014 (Ecotone, Gdansk, Poland; 35 x 16 x 12 mm, 4.2 g, 2.9% of the average little auk weight). GPSs recorded positions at 15 min intervals, and were either downloaded remotely using a base station placed in the colony, or upon recatching birds. In 2014, 3 TDR types were used: DST micro-TD (Star Oddi, Iceland; 25.4 x 8.3 mm, 3.3 g, 2.2% of the average little auk weight); LULs (CNRS, France, 17 x 9 x 5 mm, 2.2 g, 1.4% of the average little auk weight) and G5 (CEFAS Technology Limited, Lowestoft, UK, 8 x 31 mm, 2.6 g, 1.7% of the average little auk weight). In 2012, only DST micro-TD were used. Sampling intervals for both pressure and temperature were 4 s (DST micro-TD), 2 s (G5) or 1 s (LUL). Chick age was not known for equipped birds due to the difficulty to find accessible nests and nest attendance was not monitored by direct observation to limit disturbance near the nests.

#### At sea survey

In order to sample little auk prey and to assess the spatial distribution of foraging birds, an at-sea survey was conducted onboard *SV* Argelvor between August, 16–18 2014, towards the end of the chick-rearing period. No sea ice was encountered during the whole survey. Two transects were performed, one at the latitude of the colony and the other 0.29° further North, from the coast to 135 km offshore. Along each transect, 10 plankton samples were collected using a WP2 net (diameter 57 cm, mesh size 100 μm). Vertical net hauls were performed from 50 m depth to the surface with a manual winch at constant speed (mean maximum depth of birds equipped with TDRs in 2014 was 21.1 ± 4.2 m, Table 1B). Fifty meters correspond to the maximum dive depth little auks are known to forage to [30]. A Conductivity Temperature Depth sonde (CTD model YSI 600 XLM, Yellow Spring, Ohio, USA) was initially deployed above the net but did not work. Instead, a TDR (G5) was attached to the net to validate depth profiles, but temperature data could not be used to detect water masses, due to the slow response of the temperature sensor compared to net vertical speed. Zooplankton samples were stored in 70% ethanol.

**Table 1:** Statistics summary for (A) foraging trips performed by little auks equipped with GPSs (2012 and 2014); and (B) dives performed by little auks equipped with TDRs (2012 and 2014).

**A. Foraging trips**		
	**GPS**	
	**2012**	**2014**
**Number of individuals**	6	6
**Number of trips**	8	6
**Maximum distance to the colony (km, means ± SD)**	88.7 ±26.3	108.2 ±25.5
**Trip duration (hour, means ± SD)**	30.3 ±14.5	33.7 ±15.4
**B. Dives**		
	**TDR**	
	**2012**	**2014**
**Maximum dive depth (m, means ± SD)**	17.4±3.3	21.1±4.2
**Dive duration (sec, means ± SD)**	54.7 ±4.2	59.5 ±7.2
**Number of dives/24h (means ± SD)**	270 ±90	270 ±77

Bird count protocols followed Karnovsky et al [13]. All birds were counted within a 300 m radius and at 90° angle from the bow, on the one side of the boat with the best visibility when the boat was sailing. Counts stopped during net hauls. All bird species were counted but only little auk sightings are analyzed in this study.

### Data processing and analyses

#### Sea ice remote-sensing data

**Fig 2:**
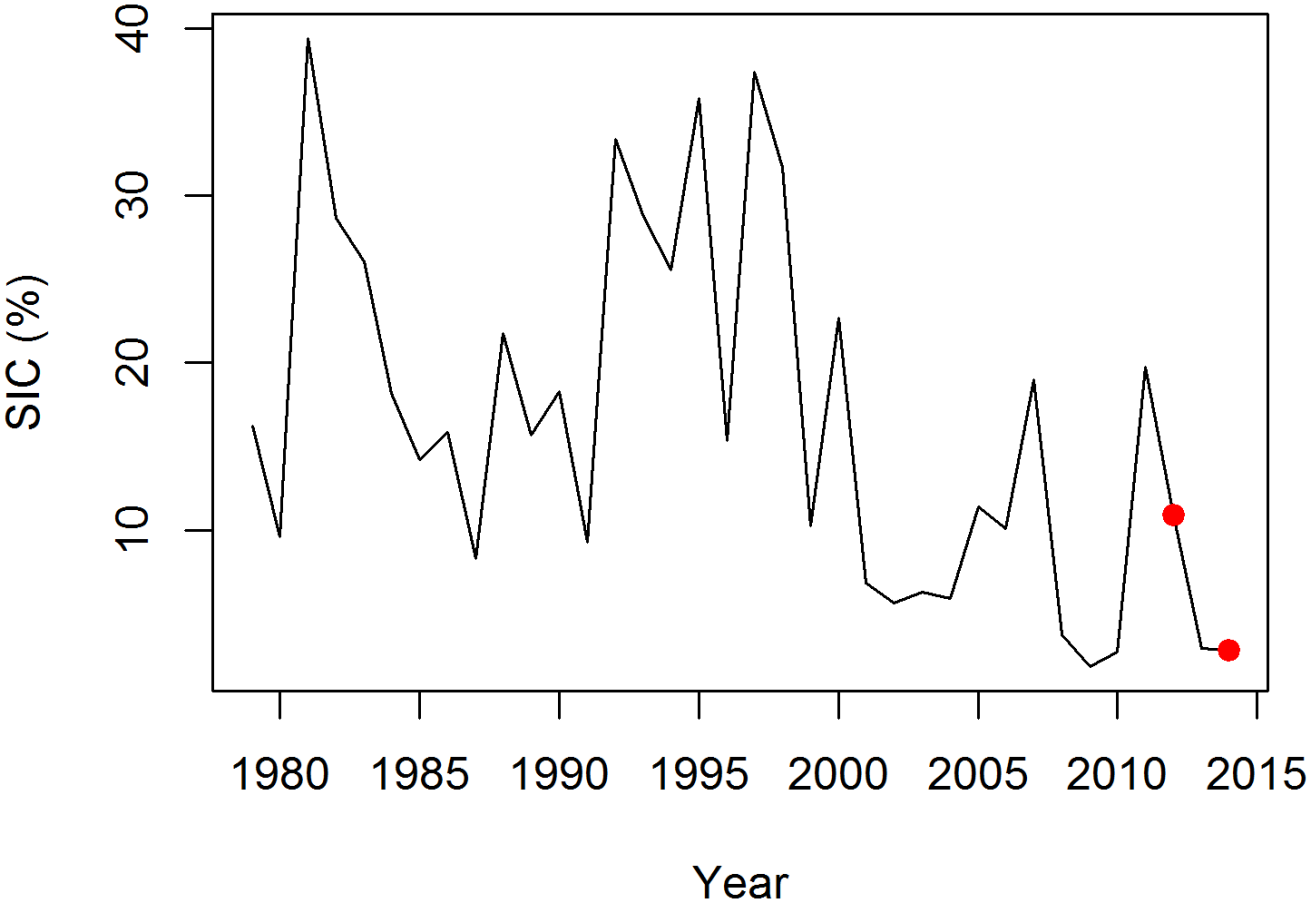
Mean sea ice concentration (SIC) in a 150x200 km area off Ukaleqarteq, East Greenland. The mean value was calculated for each year between 15 July and 15 August across 1979–2014 (reprocessed sea ice concentration dataset of the EUMETSAT OSI SAF). Red dots indicate mean SIC values for 2012 and 2014.

Daily sea ice concentrations were downloaded from the Eumetsat OSI SAF website (Eumetsat, 2011, http://osisaf.met.no/). Data from the Global Sea Ice Concentration reprocessing dataset, with a grid resolution of 12.5km, were used for the period 1978–2014 (Fig 2). An area of 150 x 200 km was determined around the colony, which included all available little auk GPS tracks. For each year, the mean sea ice concentration was calculated for this area between 15 July and 15 August. This time span contains the entire little auk chick-rearing period. For clarity, we presented sea ice extent on the maps instead of sea ice concentrations to avoid using a raster format. Daily sea ice extent data presented on the maps were downloaded from the U.S. National Ice Center (http://www.natice.noaa.gov/products/daily_products.html) and for each map, we presented the daily ice extent on the day for which we had more tracks recorded.

#### Logger data

GPS data were analyzed following Jakubas et al. [16] to determine foraging trip duration, foraging path length, and to identify positions associated to active foraging or resting. Only complete trips were used for path length and trip duration calculations. Foraging/resting areas were defined as areas were instant speed was < 10 km.h^-1^ [16]. Since both resting and foraging occurred at low speeds and could not be distinguished, we only used a single category for both behavioural patterns, which are nonetheless strongly linked since previous investigations showed that foraging birds rest at sea in the vicinity of their feeding spots (e.g. Fig 4 in [16]). For each foraging/resting position, we calculated the distance to the colony and the distance to the ice, i.e. the closest distance to the daily 80% sea-ice concentration area, obtained from the sea-ice extent maps of the U.S. National Ice Center. For each year, a kernel density estimation of foraging and resting positions was performed using the ‘adehabitatLT’ package in R [31] and a UTM 27N/GR96 projection. The smoothing parameter was calculated with the ad-hoc method and a 75% contour was chosen to represent the core foraging/resting area. Similarities of the foraging areas and the use of the shelf break were respectively quantified with the percentage of overlap between both kernels and between each kernel and the shelf-break area (defined as the area comprised between the 500 and 1500m isobaths).

TDR data were analysed using MultiTrace (Jensen Software Systems, Germany). Dive duration, maximum dive depth and the number of foraging dives per 24h were measured. To compare depth data from different TDR types, we recalibrated them in a pressure chamber. We found that DST micro-TD underestimated real depth and the following correction was therefore applied on depth data before analyses: Depth = 1.0473 * Depth(Star Oddi) + 0.4498 (1).

#### Zooplankton data

Zooplankton sampled at sea and in gular pouches was identified under a stereo microscope, to the lowest-possible taxonomic level using [32–35]. Calanus species were determined with prosome length as follows: individuals were classified by stage, photographed with the microscope-adapted camera and measured with the Image J software (U. S. National Institutes of Health, Maryland, USA). Size thresholds were defined for each stage using the size distribution obtained with our dataset.

To compare species composition between the different at-sea sampling locations, the Bray-Curtis distance was calculated and a classification tree was constructed using Ward’s method in the R package ‘Vegan’ [36,37]. We also calculated the density (individuals per m^3^) of the 3 Calanus species over the continental shelf, the shelf break and the open ocean.

The linear food selection index (LFSI) was calculated as LFSI = GP_i_−E_i_, which is the difference between the relative abundance of prey i in the gular pouch GP_i_, and in the environment E_i_ [38]. This index ranges between -1 and 1, with positive values indicating preference, and negative or null values indicating avoidance or unavailability. Mean values for LFSI, and their confidence intervals, were obtained by bootstraping 10,000 LFSI values from random GP_i_ and E_i_ which were assumed to have a normal distribution of observed means and standard deviations.

#### Chick growth and adult condition

We compared chick growth during the linear growth period (age 4–14 days, S2 Fig). A linear mixed effect model was used with mass as response variable, chick identity as a random effect and year and chick age as fixed effects. The R package ‘nlme’ was used and a model selection process using AIC was performed to select the most parsimonious model among all possible combinations of factors [39,40]. When the difference of AIC was ≤2, the model with the smallest degree of freedom was retained (S1 and S2 Tables).

An index of adult body condition was calculated following Harding et al [41]. Mass was corrected with wing length and head-bill length to take bird size into account. The index was calculated for 65 and 120 birds in 2012 and 2014, respectively. An ANCOVA was performed to test differences in residual mass between years.

## Results

### Sea ice concentration

Mean sea ice concentration in the vicinity of the little auk colony was calculated each year between July 15 and August 15 corresponding to the little auk chick rearing period. Sea ice concentration between 1979 and 2014 showed high interannual variability, with a maximum of 39.4% in 1981 and a minimum of 1.9% in 2009 (Fig 2), and declined across 1979–2014 (slope: -0.499, t(35) = -3.243, p = 0.003). In 2012, sea-ice concentration was higher than in 2014 (10.89% and 2.82%, respectively; Fig 2) and pack ice was closer to the bird colony (approx. 120 km and 300 km, respectively; Fig 1). Importantly, our at-sea surveys conducted in summer 2014 showed that the surveyed area was completely sea-ice free at the end of the chick rearing period, thereby confirming remote-sensing data.

### Foraging behaviour in relation to sea ice concentration and bathymetry

In 2012, 25 GPSs were deployed between July 19–31. Eleven tracks were recorded, with 8 complete long foraging trips from 6 birds. In 2014, 14 GPSs were deployed between July 22 and August 1. Data were recorded for 8 birds, with 6 complete foraging trips from 6 birds (Fig 1B and 1C). In total, 17 and 4 GPS-equipped loggers were recaptured in 2012 and 2014 respectively. Three more tracks were recorded in 2011 and are presented in appendix (S1 Fig).

**Fig 3:**
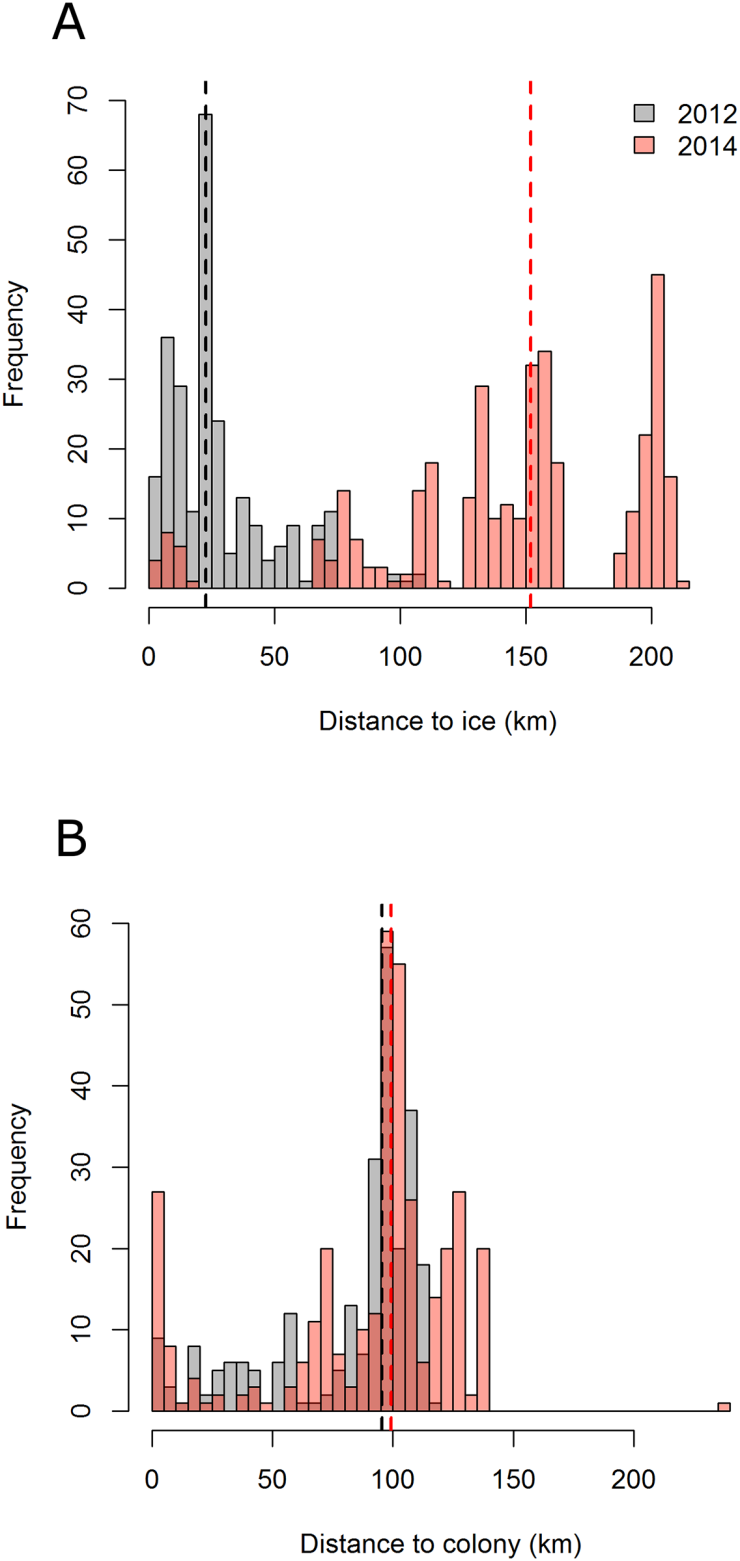
Histograms of the distance to (A) the ice (80% sea ice concentration) and (B) to the colony for foraging and resting positions in 2012 (grey) and 2014 (red). Vertical bars correspond to median values for each year.

All 15 complete GPS tracks showed a similar pattern, with unidirectional eastward commuting flights towards feeding areas. Return flights to the colony were also highly directional, with foraging/resting behaviour occurring on the way (Fig 1). Foraging/resting birds were closer to sea ice in 2012 than in 2014 (median distances of 23 and 152 km respectively, Fig 3A). However, the maximum distance from the colony reached during a foraging trip remained similar across years (89 ± 26 km in 2012 and 108 ± 26 km in 2014 (means ± SD, Table 1A, Fig 3B), and foraging/resting areas consistently overlapped with the shelf break (Fig 1). Indeed, in 2012 and 2014 the 75% kernel area of foraging/resting positions respectively overlapped by 40.4% and 42.5% with the shelf break (Fig 1D). In addition, total distance travelled, maximum distance to the colony and trip duration were not significantly different between years (Wilcoxon test: W = 19, p = 0.57; W = 15, p = 0.28 and W = 20, p = 0.66, respectively). Finally, foraging/resting kernels for 2012 and 2014 showed a 45.6% mutual overlap (Fig 1D). Three birds equipped in 2011, in the presence of sea ice, performed similar trips as birds equipped in 2012 and 2014 (S1 Fig).

In 2012, 13 TDRs were deployed on August 2. Nine were retrieved and one file was corrupted. In 2014, 18 TDRs were deployed between July 20 and August 8. Twelve of them were retrieved and 7 recordings were used for the analyses. Diving behaviour (maximum dive depth, dive duration and the number of dives per 24h) did not differ between years (Table 1B, Wilcoxon test: W = 42, p = 0.12; W = 43, p = 0.094; W = 29, p = 0.96 respectively).

**Fig 4:**
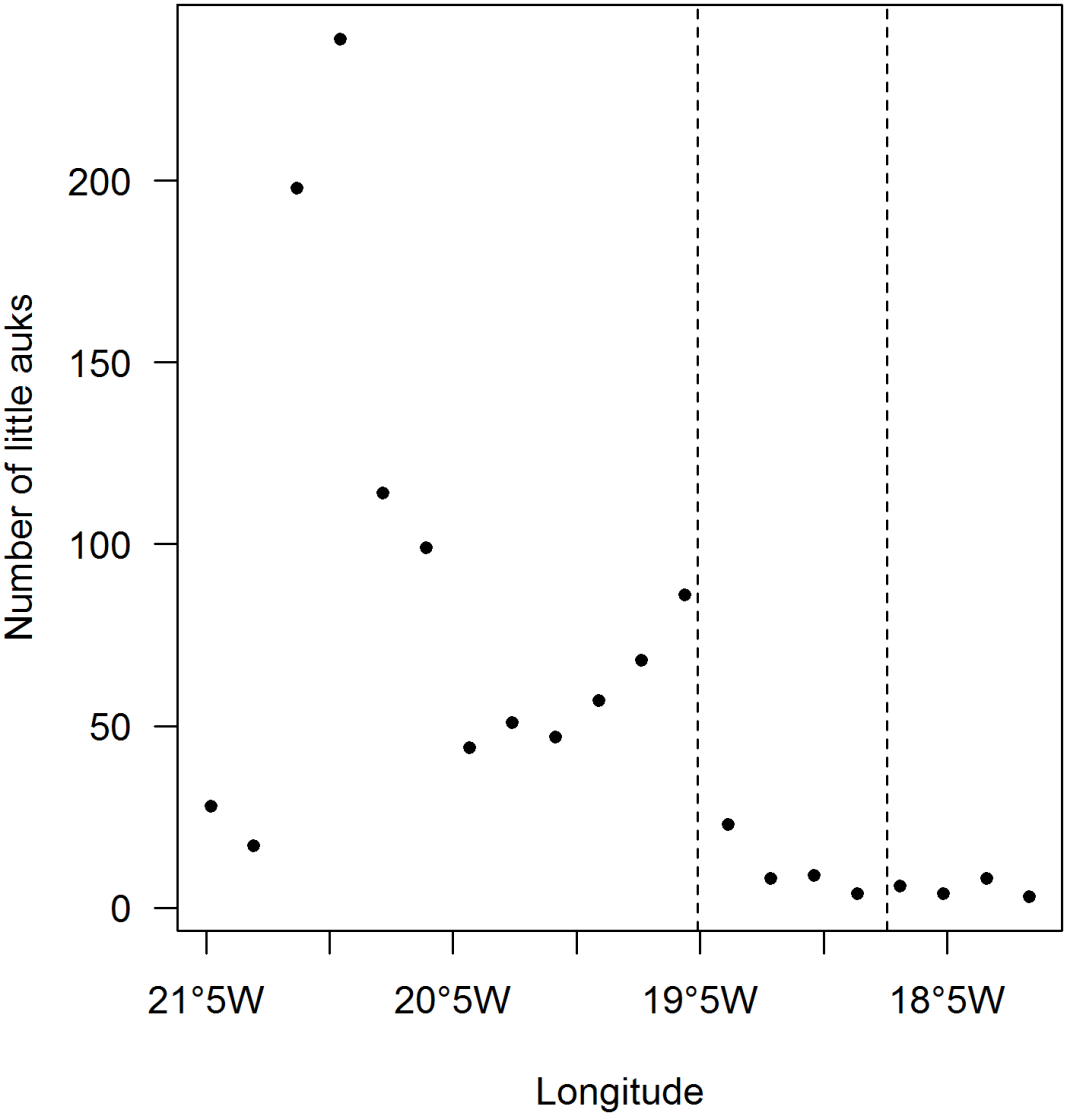
At-sea little auk sightings in 2014, in the absence of sea ice. Data from the two transects (Fig 5) were pooled and counts were grouped according to longitude. The two dashed lines represent the longitude of the 500 m and 1500 m isobaths, which delimit the shelf break.

At-sea counts of little auks performed in 2014, in the absence of sea ice, showed a first peak of abundance on the continental shelf (Fig 4) and a second lower peak close to the 500 m isobath, which corresponds to the beginning of the shelf break (Fig 4).

### Chick diet

The composition of prey brought back to chicks by adults differed between years (Table 2). Calanoid copepods (*Calanus sp*.) were the main prey type in both years (85% in 2012 and 90% in 2014), but proportion of the three *Calanus* species present in the diet differed between years. The largest species *C*. *hyperboreus* was more abundant in 2012 (t(38) = 2.43, p = 0.02), whereas the smaller *C*. *glacialis* and *C*. *finmarchicus* were more abundant in 2014 (t-tests, t(38) = -5.28, p < 0.001 and t(38) = -2.75, p < 0.001 respectively). Further, ice-associated amphipods represented nearly 10% of the diet in 2012 and were virtually absent in 2014. In particular, the amphipod *Apherusa glacialis* represented up to 96% of single food loads in 2012, and only a few single specimens were found in 2014. The linear food selection index (LFSI, [38]) indicated which prey items were preferentially collected by parent little auks in 2014 (Table 2): LFSI was the highest for *Calanus hyperboreus* and *C*. *glacialis* (LFSI = 0.43 and 0.28 respectively, Table 2) indicating that they were actively selected by birds. Other prey species had low or negative indexes, indicating that they were avoided or opportunistically taken. In particular, *Calanus finmarchichus* LFSI was not different from 0 despite its high abundance in the environment and was therefore not selected (Table 2). Small *Calanus* copepodite stages (I-III) were also avoided by parent little auks to feed their chick (negative LFSI, Table 2).

**Table 2:** Relative abundance (RA, mean ± SD, %) and occurrence frequency (OF, %) of zooplankton found in little auk gular pouches in 2012 and 2014, and in at-sea samples collected in 2014; and linear food selection index (mean ± SD, %) for 2014 prey. *Calanus hyperboreus*, *C*. *glacialis* and *C*. *finmarchicus* groups included the stages CIV, CV, and adult males and females. Other copepodite stages of these 3 species were included in the ‘*Calanus* CI-CIII’ group. Species included in the ‘small copepods’ group are *Triconia borealis*, *Scaphocalanus magnus*, *Metridia longa* and *Microcalanus spp* for the Continental shelf, and *Triconia borealis*, *Metridia longa* and *Microcalanus spp* for the shelf break and the open ocean. Main little auk prey species are in bold. Asterisk indicates ice-associated prey. Continental shelf, shelf break and open ocean groups were defined based on isobaths (< 500 m, 500–1500 m and > 1500 m respectively). Linear food selection index is the difference between prey proportion found in little auk gular pouch and prey proportion in the environment and ranges from -1 to 1 [38]. A positive value indicates preference and negative or null values avoidance or unavailability.

	Little auk gular pouch				At sea samples 2014						Linear food selection index		
	2012		2014		Continental shelf		Shelf break		Open ocean		2014		
	RA	OF	RA	OF	RA	OF	RA	OF	RA	OF	Continental shelf	Shelf break	Open ocean
**C. hyperboreus**	**65.3 ± 28.6**	**90**	**47.0 ± 17.6**	**100**	**3.9 ± 2.7**	**100**	**0.4 ± 0.8**	**40**	**0.2 ± 0.3**	**50**	**0.43 ± 0.18**	**0.46 ± 0.18**	**0.46 ± 0.18**
**C. glacialis**	**15.5 ± 9.2**	**95**	**34.1 ± 12.8**	**100**	**6.3 ± 3.1**	**100**	**1.8 ± 3.2**	**80**	**0.6 ± 0.8**	**75**	**0.28 ± 0.13**	**0.32 ± 0.13**	**0.334 ± 0.13**
**C. finmarchicus**	**4.2 ± 4.0**	**100**	**9.0 ± 6.7**	**100**	**4.8 ± 3.2**	**100**	**7.6 ± 5.0**	**100**	**10.1 ± 6.5**	**100**	**0.04 ± 0.07**	**0.02 ± 0.08**	**-0.01 ± 0.09**
Calanus CI-CIII	0.0 ± 0.0	0	0.0 ± 0.0	0	38.6 ± 8.4	100	26.4 ± 17.1	100	15.5 ± 1.2	100	-0.38 ± 0.08	-0.27 ± 0.17	-0.16 ± 0.01
Small copepods	0.0 ± 0.0	0	0.0 ± 0.0	0	16.1 ± 9.5	100	36.6 ± 15.7	100	42.4 ± 4.4	100	-0.16 ± 0.10	-0.36 ± 0.16	-0.42 ± 0.04
Paraeuchaeta spp	0.01 ± 0.05	5	0.1 ± 0.3	15	0.0 ± 0.0	0	0.0 ± 0.0	0	0.0 ± 0.0	0	0.0 ± 0.0	0.0 ± 0.0	0.0 ± 0.0
Oithona spp	0.0 ± 0.0	0	0.0 ± 0.0	0	8.6 ± 3.9	100	11.0 ± 5.3	100	10.5 ± 6.9	100	-0.09 ± 0.04	-0.11 ± 0.05	-0.11 ± 0.07
Apherusa glacialis*	**8.7 ± 26.4**	**45**	**0.1 ± 0.3**	**20**	**0.0 ± 0.0**	**0**	**0.0 ± 0.0**	**0**	**0.0 ± 0.0**	**0**	**0.0 ± 0.0**	**0.0 ± 0.0**	**0.0 ± 0.0**
Gammarus spp*	0.5 ± 1.9	40	0.0 ± 0.0	0	0.0 ± 0.0	0	0.0 ± 0.0	0	0.0 ± 0.0	0	0.0 ± 0.0	0.0 ± 0.0	0.0 ± 0.0
Onisimus spp*	0.3 ± 0.7	40	0.0 ± 0.0	0	0.0 ± 0.0	0	0.0 ± 0.0	0	0.0 ± 0.0	0	0.0 ± 0.0	0.0 ± 0.0	0.0 ± 0.0
Thysanoessa spp	4.2 ± 13.8	15	4.7 ± 11.4	30	0.0 ± 0.0	0	0.0 ± 0.0	0	0.0 ± 0.0	0	0.05 ± 0.11	0.05 ± 0.11	0.05 ± 0.11
Themisto spp	1.1 ± 1.6	55	4.2 ± 5.2	90	0.1 ± 0.2	36	0.1 ± 0.1	60	0.1 ± 0.1	75	0.04 ± 0.05	0.04 ± 0.05	0.04 ± 0.05
Decapod larvae	0.01 ± 0.03	5	0.1 ± 0.3	10	0.0 ± 0.0	0	0.0 ± 0.0	0	0.0 ± 0.0	0	0.0 ± 0.0	0.0 ± 0.0	0.0 ± 0.0
Fish	0.03 ± 0.1	10	0.2 ± 0.3	30	0.0 ± 0.0	0	0.0 ± 0.0	0	0.0 ± 0.0	0	0.0 ± 0.0	0.0 ± 0.0	0.0 ± 0.0
Limacina helicina	0.2 ± 0.4	25	0.1 ± 0.4	10	1.1 ± 1.0	100	0.1 ± 0.1	20	0.0 ± 0.0	0	-0.01 ± 0.01	0.0 ± 0.0	0.0 ± 0.0
Nauplius larvae	0.0 ± 0.0	0	0.0 ± 0.0	0	4.3 ± 3.0	91	6.2 ± 4.8	100	2.8 ± 1.0	100	-0.04 ± 0.03	-0.06 ± 0.05	-0.03 ± 0.01
Tunicates	0.0 ± 0.0	0	0.0 ± 0.0	0	6.2 ± 2.9	100	4.1 ± 2.9	100	7.3 ± 2.3	100	-0.06 ± 0.03	-0.04 ± 0.03	-0.07 ± 0.02
Echinodermata larvae	0.0 ± 0.0	0	0.0 ± 0.0	0	5.9 ± 6.4	91	0.6 ± 0.3	100	0.3 ± 0.3	75	-0.06 ± 0.06	-0.01 ± 0.00	0.0 ± 0.0
Unknown	0.01 ± 0.02	5	0.1 ± 0.3	5	4.2 ± 2.1	100	5.2 ± 2.1	100	10.2 ± 3.9	100	-0.04 ± 0.02	-0.05 ± 0.02	-0.10 ± 0.04

### Chick growth and adult body condition

Chick mass was monitored for 24 chicks in 2012 and 29 chicks in 2014 (S2 Fig). The linear growth period was modelled using a linear mixed effect model including chick age and year, and chick ID was added as a random factor (S1 Table). Chick age was the only relevant factor in the selected model (S1 and S2 Tables). Year was rejected during model selection process, indicating that chick growth did not differ between years (S2 Fig). Adult body condition was calculated from 65 and 120 birds in 2012 and 2014, respectively. No difference in adult body condition was found between years (ANCOVA, F_1,183_ = 0.064, p = 0.8, S3 Fig).

### Bathymetry impact on zooplankton community composition

**Fig 5:**
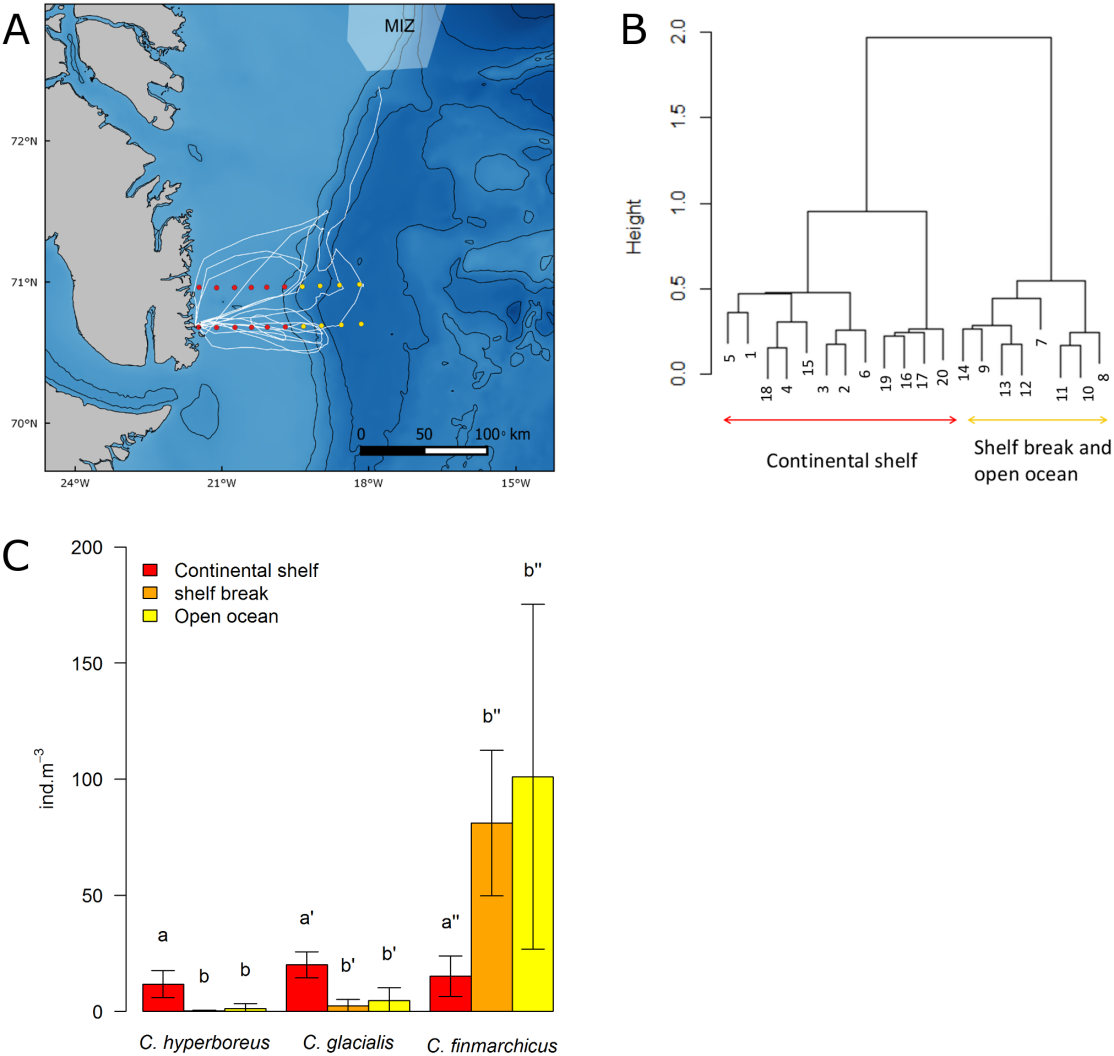
Zooplankton communities sampled along two transects in 2014. (A) Location of zooplankton sampling transects, little auk GPS tracks for 2014 (white line), bathymetry (black lines: 500-m isobaths) and sea ice extent (17 August 2014, only the marginal ice zone (MIZ) is present). (B) Cluster dendrogram of zooplankton species composition grouped according to Bray-Curtis distance. This allowed associating samples to a ‘continental shelf’ (red) or ‘open ocean’ (yellow) community, and those groups/colours are reported on Fig 5A. (C) Number of individuals per m^3^ of the 3 Copepod species in the 3 marine habitats defined by bathymetry. For each species, means with different letters are significantly different (Wilcoxon test, p<0.01).

During our 2014 at-sea survey, 20 zooplankton samples were collected on two transects across the shelf break, corresponding to little auk foraging areas (Fig 5A). Given the Bray-Curtis distance measuring dissimilarity between samples, zooplankton composition was grouped in two communities (Fig 5B). The first included samples collected on the continental shelf, and the second samples from the shelf break and the open ocean (Fig 5). The density of *Calanus* species depended on bathymetric features (Fig 5C): *C*. *glacialis* and *C*. *hyperboreus* (main prey items found in little auk chick diets, Table 2) were both more abundant on the continental shelf than on the shelf break and the open ocean (Kruskal-Wallis test, p < 0.01 for both species, Fig 5C). On the contrary, *C*. *finmarchicus* was more abundant on the shelf break and the open ocean, with densities nearly 5 times higher than for each of the 3 species on the continental shelf (Kruskal-Wallis test, p < 0.01, Fig 5C).

## Discussion

During their foraging trips, little auks visited similar areas of the continental shelf and of the shelf break, irrespective of the presence/absence of sea-ice (Fig 1 and S1 Fig). Chick growth curves and adult body mass (S2 and S3 Figs) were also similar across years with/without sea ice, suggesting unaffected reproductive output and adult body condition. We conclude that bathymetry is potentially more important to foraging little auks than sea ice. Our results are particularly relevant in the context of Arctic climate change, and of the environmental impacts of vanishing sea ice cover.

### Bathymetry effects

Information provided by GPSs and at-sea observations show that little auks foraged preferentially on the shelf break and on the continental shelf (Figs 1, 4 and S1). Upwelling areas or fronts are known to occur along shelf breaks and to concentrate seabird prey [23,42]. In the studied area, the East Greenland break front could play this role [43]. Many examples show seabird association with shelf breaks worldwide, among which Cory’s shearwaters *Calonectris diomedea* in the Mediterranean Sea [44], black petrels *Procellaria parkinsoni* off New Zealand [45], or black-footed albatrosses *Phoebastria nigripes* on the Californian shelf slope [46]. In addition, numerous studies have mentioned the presence of Alcids at fronts, among which Murres (*Uria lomvia* and *U*. *aalge*) in the East Bering Sea [47], and planktivorous auklets taking advantage of tidal fronts at the sills between the Aleutian islands [48,49]. Concerning little auks, one exception has been highlighted by Karnovsky and collaborators. They found that little auks from Hornsund (South-West Spitsbergen) foraged mainly on the continental shelf (Arctic water from the Sørkapp Current) but avoided the shelf break where they encountered waters of Atlantic origin (West Spitsbergen Current) [12]. This difference with our results was most likely due to prey types and densities. Off Hornsund, small and less profitable prey species were in the same densities within Atlantic water masses than the bigger and richer ones within Arctic water masses, and were further away from the colony. Little auks therefore had no incentives to forage at the shelf break. In contrast, in our system in the absence of ice, smaller species were largely more abundant at the shelf break than the bigger ones upon the shelf area (Fig 5).

In 2014, chick diets were mainly composed of *C*. *hyperboreus* and *C*. *glacialis* (Table 2) and these species were more abundant on the continental shelf (Fig 5C). However, 42.5% of adult foraging/resting areas were situated on the shelf break. There, *C*. *finmarchicus* densities were about five times greater than densities found on the continental shelf for the three copepod species. This comparison between chick diet, adult foraging/resting areas and Calanus copepod densities suggests that breeding little auks may not feed on the same prey and in the same areas when foraging for themselves, or for their chicks as described by Brown and collaborators [50]. More specifically, it suggests that breeding birds fed for themselves first. Tracked birds targeted the shelf break, probably to feed for themselves on highly abundant, yet smaller and less calorific *C*. *finmarchicus*. Then, on their way back across the continental shelf, they likely caught *C*. *hyperboreus* and *C*. *glacialis* for their chicks. These two copepods are less abundant but larger and more calorific than *C*. *finmarchicus* [20]. This hypothesis is consistent with the fact that most adult little auks caught at colonies have empty stomachs [51] and that nearly all studies focusing on adult diet were performed on birds caught at sea [14,17,18,51,52]. It is therefore particularly difficult to compare adult and chick diet [51]. Previous isotopic investigations performed at the same colony suggested that breeding adults and their chicks feed at the same trophic level [53]. However, and because the three copepod species are all mainly herbivorous and present similar isotopic signatures/trophic positions [54,55], this method cannot be used to confirm our hypothesis, and further investigations are required to confirm factors driving the combined use of continental shelf and shelf break by foraging parent little auks.

### Sea ice effects

In the present study, little auks foraged in the same areas in the presence and nearby absence of sea ice (Fig 1 and S1 Fig), thus questioning the importance of sea-ice habitats for foraging little auks during the breeding season [56]. Previous studies led in West Spitsbergen found that little auks foraged mainly in the marginal ice zone and could modulate their foraging distance to track sea ice up to 150 km [16,57]. However, areas where little auks foraged in Spitsbergen were also close to the shelf break where the Arctic front separates Atlantic waters from the West Spitsbergen current and Arctic waters from the Sørkapp current [58]. It is thus possible that bathymetry also played an important role for foraging little auks in Spitsbergen, as in East Greenland. However, the spatial match between the shelf break and the marginal ice zone in both Spitsbergen and East Greenland precludes further quantitative evaluation of the role played by each parameter, and further studies from other arctic areas are needed.

In our system, another open question is whether little auks could cope with longer foraging trips to reach sea ice areas. Going as close to the ice in 2014 as in 2012 (i.e. travelling an additional 258 km, Fig 3) would have required little auks to increase their flight times by six hours per foraging trip, with an overall 18% increase in trip duration. Compared with other published data on foraging trip duration or maximum foraging distance, little auks from our study already performed trips which were longer (mean duration of 15.9 h and 22.7 h [59,60]; mean maximum distance of 67.1 km and 65.5 km [57,60]) or of the same order of magnitude (mean maximum distance of 97.9 km and mean duration of 24.3 h, [16]) than little auks from other areas. This suggests that birds may have already been operating to their maximum capabilities, and probably cannot reach the ice when it is further away. Further, our two study years actually represent moderate sea ice conditions for little auks in East Greenland. Indeed, 2012 and 2014 are within the lower range of sea ice extents over the 1979–2014 period (Fig 2), and the current sea ice decline started well before the advent of satellite measurements [61]. We can therefore only speculate about the foraging behaviour of little auks exposed to substantially higher sea ice concentrations > 20 years ago. Under these conditions, understanding which other parameters influence little auk foraging behaviour, such as bathymetry, is crucial to anticipate their response to an ice-free environment.

Further, sea ice is also important for little auks and other Arctic seabirds as a platform to rest during foraging trips [14], and its disappearance may increase the energetic costs of resting. In spectacled eiders (*Somateria fischeri*), for instance, resting at the sea surface was estimated to be 50% more costly, energetically, than resting on sea ice [62] and the same trend is expected for other seabird species [2]. This could alter adult body condition, especially in little auks that are known to have an elevated metabolism [20]. In our study, it is possible that some physical processes aggregated small remaining ice floes at the shelf break in 2014 and that these platforms were used for birds to digest food and rest in the middle of their trip as we could not distinguish resting and foraging behaviour.

### Device effects

Birds equipped with GPSs showed significantly longer trip durations when compared with literature data for the same colony (mean long trip duration of 9.6 ± 0.5 h, obtained with direct observations of marked, yet unequipped birds in 2007 [59]). Moreover, birds from these two studies were investigated a different times of the chick-rearing phases, and hence it cannot be excluded that birds modified their foraging behaviour as the season progressed, or that prey availability and foraging conditions differed between years [57]. However, a similar bias towards longer foraging trips was also observed in little auks equipped with the same type of GPS devices in Spitsbergen [16]. Devices attached to diving seabirds increase hydrodynamic drag [63], and has been shown to affect trip duration in some species, including little auks [64,65]. Overall, we recommend that future little auk GPS-tracking studies use smaller/lighter tags, to ensure the smallest possible impact on the birds. Nevertheless, as birds equipped with GPSs in the presence/absence of sea ice in 2012 and 2014 were similarly handicapped by tags, we consider that our comparison of their utilization of the marginal ice zone and of bathymetric features during foraging trips remains valid.

### Little auks and climate change

Climate change may affect arctic seabirds through (1) changes in the behaviour of their predators. For instance, enhanced polar bear predation on seabirds has been demonstrated in recent years [66], see also [67]. (2) The spread of new parasites and pathogens from lower latitudes (e.g. [68,69]), and (3) changes in their breeding and foraging habitats. Notably, former work showed that little auks perform better in cold Arctic waters containing lipid-rich copepods [10,13,70]. In the longer term, an increase in sea surface temperature in the Arctic is therefore expected to favour boreal copepods such as *Calanus finmarchicus* [71]. *C*. *finmarchicus* are smaller and contain less lipids than the two Arctic copepods *C*. *glacialis* and *C*. *hyperboreus*. Moreover, higher temperatures are expected to favour smaller zooplankton, both smaller species and smaller individuals within a species [72,73]. Such a negative relationship between organismal size and temperature was observed during geological times for phytoplankton [74]. While adult little auks might be able to cope with smaller prey like *C*. *finmarchicus* in their environment (this study, [75]), the food selection index showed that breeding birds had a strong preference for large copepods to feed their chick (Table 2). This finding raises the questions whether, in the longer term, adult little auks will have capabilities to gather more/enough smaller and less profitable prey to meet their chick energetic requirements and whether chicks will deal with smaller prey in their diet. One study indeed found that when there was a larger influx of Atlantic water off Hornsund, Spitsbergen, chick meals were of lower mass and lower energy content, and parents had to increase the number of foraging trips to fulfil chick dietary needs [76].

Comparative studies conducted across the Greenland Sea do indicate that little auks have for now the capacity to buffer the consequences of current ocean warming, through marked foraging plasticity [10,76]. Further, recent investigations demonstrated that, in the absence of sea-ice, little auks may efficiently switch from offshore feeding habitats to less distant, prey-rich coastal fronts created by the melt water of retreating coastal glaciers [77], and unexpectedly, may efficiently feed in warm Atlantic waters containing boreal zooplankton species at their southernmost breeding site [60]. These results confirm that little auks are so far flexible with respect to the consequences of arctic warming, challenging current species distribution models forecasting future distributions for little auks in a warming Arctic [13,78]. Crucially, our study strongly suggests that bathymetric features and associated productivity may actually be more important for efficient little auk foraging than sea-ice habitats. Nevertheless, these ideas should now be tested using smaller tracking devices, and at further study sites, to fully understand the importance of bathymetry for foraging little auks across the Arctic.

## Supporting Information

S1 Fig GPS tracks of 3 little auks in 2011 and sea ice extent. Red dots correspond to foraging or resting (speed <10 km.h^-1^) and black dots to travelling (instant speed >10 km.h^-1^). Sea ice extent data were downloaded from the U.S. National Ice Center (http://www.natice.noaa.gov/products/daily_products.html). White: pack ice with an ice concentration >80%. Light blue: marginal ice zone (MIZ) with an ice concentration <80%. In the marginal ice zone, sea ice concentration decreased between pack ice and open water. Only 1 out of 4 trips were complete, thus we did not include these tracks in our analyses. For the complete track, the maximum distance to the colony was 84.3 km and the trip duration was 22.4h. Projection: GR96/ UTM zone 27N.
(PDF)

S2 Fig Chick Growth curves for 2012 (black, n = 24 chicks) and 2014 (red, n = 29 chicks). Chicks were weighed every second day. Chick growth was compared between years during the linear growth period (delimited by dotted lines) using a linear mixed effect model with mass as an explanatory variable, chick age and year as fixed factors and chick as a random factor. Model selection process retained model with chick age as fixed factor and chick as random effect. Year factor was rejected meaning that there is no difference of chick growth between years.
(PDF)

S3 Fig Adult body condition index (mean±SE) in 2012 (black, n = 65) and 2014 (red, n = 120) calculated following [42]. There was no difference in body condition between both years (F_1,183_ = 0.064, p = 0.8).
(PDF)

S1 Table Model selection using Akaike’s information criterion (AIC) to test the effects of age and year on chick body mass (51 chicks, 256 observations). Model selection of all combination of factors, a chick random effect is included. K: number of parameters. ΔAIC is the difference of AIC between a given model and the model with the lowest AIC. Best model is number 1 with the smallest AIC and less parameters than model 2.
(PDF)

S2 Table Parameter estimation of model 1 testing the effects of age on chick body mass. (PDF)

## Abbreviations

E_i_: relative abundance of prey i in the environment
GP_i_: relative abundance of prey i in gular pouch
LFSI: linear food selection index
MIZ: Marginal ice zone, transition area between pack ice and the open ocean. Defined by a sea ice concentration <80% on our figures
Pack ice: also called “drift ice”, sea ice that is not attached to land. If attached to land it is called fast ice
SIC: sea ice concentration, percentage of sea surface covered by ice in a given area
TDR: temperature depth recorder

## References

[1] IPCC. Climate Change 2013: The Physical Science Basis Contribution of Working Group I to the Fifth Assessment Report of the Intergovernmental Panel on Climate Change. [Stocker T.F., Qin D., Plattner G.-K., Tignor M., Allen S.K., Boschung J., Nauels A., Xia Y., Bex V. and Midgley P.M. (eds.)] Cambridge University Press, Cambridge, United Kingdom and New York, NY, USA, 1535 pp. 2013.

[2] Eamer J, Donaldson G, Gaston T, Kosobokova K, Lárusson KF, Melnikov I, et al Life linked to ice. A guide to sea-ice associated biodiversity in this time of rapid change Conservation of Arctic Flora and Fauna (CAFF) Akureyri, Iceland Akureyri, Iceland: Conservation of Arctic Flora and Fauna (CAFF); 2013.

[3] Arndt CE, Swadling KM. Crustacea in Arctic and Antarctic sea ice: distribution, diet and life history strategies. Adv Mar Biol. 2006;51: 197–315. 16905428 10.1016/S0065-2881(06)51004-1

[4] Gilg O, Kovacs KM, Aars J, Fort J, Gauthier G, Grémillet D, et al Climate change and the ecology and evolution of Arctic vertebrates. Ann N Y Acad Sci. 2012;1249: 166–190. 10.1111/j.1749-6632.2011.06412.x 22329928

[5] Søreide JE, Leu E, Berge J, Graeve M, Falk-Petersen S. Timing of blooms, algal food quality and Calanus glacialis reproduction and growth in a changing Arctic. Glob Change Biol. 2010;16: 3154–3163. 10.1111/j.1365-2486.2010.02175.x

[6] Doney SC, Ruckelshaus M, Emmett Duffy J, Barry JP, Chan F, English CA, et al Climate change impacts on marine ecosystems. Annu Rev Mar Sci. 2012;4: 11–37. 10.1146/annurev-marine-041911-111611, 22457967

[7] Post E, Bhatt US, Bitz CM, Brodie JF, Fulton TL, Hebblewhite M, et al Ecological consequences of sea-ice decline. Science. 2013;341: 519–524. 10.1126/science.1235225 23908231

[8] Wassmann P, Duarte CM, Agustí S, Sejr MK. Footprints of climate change in the Arctic marine ecosystem. Glob Change Biol. 2011;17: 1235–1249. 10.1111/j.1365-2486.2010.02311.x

[9] Egevang C, Boertmann D, Mosbech A, Tamstorf MP. Estimating colony area and population size of little auks *Alle alle* at Northumberland Island using aerial images. Polar Biol. 2003;26: 8–13.

[10] Grémillet D, Welcker J, Karnovsky NJ, Walkusz W, Hall ME, Fort J, et al Little auks buffer the impact of current Arctic climate change. Mar Ecol Prog Ser. 2012;454: 197–206.

[11] Hovinen JEH, Wojczulanis-Jakubas K, Jakubas D, Hop H, Berge J, Kidawa D, et al Fledging success of little auks in the high Arctic: do provisioning rates and the quality of foraging grounds matter? Polar Biol. 2014;37: 665–674. 10.1007/s00300-014-1466-1

[12] Karnovsky NJ, Kwasniewski S, Weslawski JM, Walkusz W, Beszczynska-Moller A. Foraging behavior of little auks in a heterogeneous environment. Mar Ecol Prog Ser. 2003;253: 289–303. 10.3354/meps253289

[13] Karnovsky N, Harding A, Walkusz W, Kwasniewski S, Goszczko I, Wiktor J, et al Foraging distributions of little auks *Alle alle* across the Greenland Sea: implications of present and future Arctic climate change. Mar Ecol Prog Ser. 2010;415: 283–293. 10.3354/meps08749

[14] Bradstreet MSW. Pelagic feeding ecology of dovekies, *Alle alle*, in Lancaster Sound and Western Baffin Bay. Arctic. 1982;35: 126–140.

[15] Jakubas D, Iliszko L, Wojczulanis-Jakubas K, Stempniewicz L. Foraging by little auks in the distant marginal sea ice zone during the chick-rearing period. Polar Biol. 2012;35: 73–81. 10.1007/s00300-011-1034-x

[16] Jakubas D, Trudnowska E, Wojczulanis-Jakubas K, Iliszko L, Kidawa D, Darecki M, et al Foraging closer to the colony leads to faster growth in little auks. Mar Ecol Prog Ser. 2013;489: 263–278. 10.3354/meps10414

[17] Lønne OJ, Gabrielsen GW. Summer diet of seabirds feeding in sea-ice-covered waters near Svalbard. Polar Biol. 1992;12: 685–692.

[18] Mehlum F. Seabird species associations and affinities to areas covered with sea ice in the northern Greenland and Barents Seas. Polar Biol. 1997;18: 116–127.

[19] Fort J, Moe B, Strøm H, Grémillet D, Welcker J, Schultner J, et al Multicolony tracking reveals potential threats to little auks wintering in the North Atlantic from marine pollution and shrinking sea ice cover. Divers Distrib. 2013;19: 1322–1332. 10.1111/ddi.12105

[20] Harding AMA, Egevang C, Walkusz W, Merkel F, Blanc S, Grémillet D. Estimating prey capture rates of a planktivorous seabird, the little auk (*Alle alle*), using diet, diving behaviour, and energy consumption. Polar Biol. 2009;32: 785–796.

[21] Parsons TR, Takahashi M, Hargrave B. Biological oceanographic processes. Elsevier; 1984.

[22] Yen PP, Sydeman WJ, Hyrenbach KD. Marine bird and cetacean associations with bathymetric habitats and shallow-water topographies: implications for trophic transfer and conservation. J Mar Syst. 2004;50: 79–99.

[23] Bost C-A, Cotté C, Bailleul F, Cherel Y, Charrassin J-B, Guinet C, et al The importance of oceanographic fronts to marine birds and mammals of the southern oceans. J Mar Syst. 2009;78: 363–376.

[24] Fifield DA, Lewis KP, Gjerdrum C, Robertson GJ, Wells R. Offshore seabird monitoring program. Environ Stud Res Funds Rep. 2009;183.

[25] Follestad A. The pelagic distribution of Little Auk *Alle alle* in relation to a frontal system off central Norway, March/April 1988. Polar Res. 1990;8: 23–28.

[26] Skov H, Durinck J. Seabird distribution in relation to hydrography in the Skagerrak. Cont Shelf Res. 2000;20: 169–187. 10.1016/S0278-4343(99)00067-9

[27] R Core Team. A language and environment for statistical computing. Vienna, Austria: R Foundation for Statistical Computing; 2013 URL Http://www.R-Proj.Org. 2013.

[28] QGIS Development Team. QGIS Geographic Information System [Internet]. Open Source Geospatial Foundation; 2015 Available: http://qgis.osgeo.org.

[29] Mosbech A, Johansen KL, Bech NI, Lyngs P, Harding AM, Egevang C, et al Inter-breeding movements of little auks *Alle alle* reveal a key post-breeding staging area in the Greenland Sea. Polar Biol. 2012;35: 305–311.

[30] Fort J, Cherel Y, Harding AM, Egevang C, Steen H, Kuntz G, et al The feeding ecology of little auks raises questions about winter zooplankton stocks in North Atlantic surface waters. Biol Lett. 2010;6: 682–684. 10.1098/rsbl.2010.0082 20236962 PMC2936134

[31] Calenge C. The package “adehabitat” for the R software: a tool for the analysis of space and habitat use by animals. Ecol Model. 2006;197: 516–519.

[32] Dunbar MJ. Amphipoda, sub-order Hyperiidea, family Hyperiidae. Zooplankton sheet 103. Cons Int Pour Explor Mer. 1963; 4.

[33] Klekowski RZ, Weslawski JM. Atlas of the marine fauna of southern Spitsbergen, vol 2, Invertebrates: Part 1 Inst Oceanol Gdansk. 1991.

[34] Rose M. Copépodes pélagiques. Paris: P. Lechevalier; 1933.

[35] Tencati JR, Leung Y, Kobayashi H. Taxonomic guides to arctic zooplankton (I): Amphipods of the central Arctic, Euphausiids of the Arctic Basin and peripheral seas. DTIC Document; 1970.

[36] Legendre P, Legendre LFJ. Numerical Ecology. Elsevier; 2012.

[37] Oksanen J, Blanchet FG, Kindt R, Oksanen MJ, Suggests M. Package “vegan.” Community Ecol Package Version. 2013;2: 0–0.

[38] Strauss RE. Reliability estimates for Ivlev’s electivity index, the forage ratio, and a proposed linear index of food selection. Trans Am Fish Soc. 1979;108: 344–352.

[39] Pinheiro J, Bates D, DebRoy S, Sarkar D, the R Development Core Team. nlme: linear and nonlinear mixed effects models R Package Version 31–111. 2013.

[40] Zuur AF, Ieno EN, Walker NJ, Saveliev AA, Smith GM. Mixed effects models and extensions in ecology with R Springer; 2009.

[41] Harding AM, Welcker J, Steen H, Hamer KC, Kitaysky AS, Fort J, et al Adverse foraging conditions may impact body mass and survival of a high Arctic seabird. Oecologia. 2011;167: 49–59. 10.1007/s00442-011-1971-7 21445685

[42] Genin A. Bio-physical coupling in the formation of zooplankton and fish aggregations over abrupt topographies. J Mar Syst. 2004;50: 3–20. 10.1016/j.jmarsys.2003.10.008

[43] Belkin IM, Cornillon PC, Sherman K. Fronts in large marine ecosystems. Prog Oceanogr. 2009;81: 223–236.

[44] Louzao M, Bécares J, Rodríguez B, Hyrenbach KD, Ruiz A, Arcos JM, et al Combining vessel-based surveys and tracking data to identify key marine areas for seabirds. Mar Ecol Prog Ser. 2009;391: 183–197.

[45] Freeman R, Dennis T, Landers T, Thompson D, Bell E, Walker M, et al Black petrels (*Procellaria parkinsoni*) patrol the ocean shelf-break: GPS tracking of a vulnerable Procellariiform seabird. PLoS ONE. 2010;5: e9236 10.1371/journal.pone.0009236 20174652 PMC2822854

[46] Hyrenbach KD, Keiper C, Allen SG, Ainley DG, Anderson DJ. Use of marine sanctuaries by far-ranging predators: commuting flights to the California Current System by breeding Hawaiian albatrosses. Fish Oceanogr. 2006;15: 95–103.

[47] Kinder TH, Hunt GL, Schneider D, Schumacher JD. Correlations between seabirds and ocenic fronts around the Pribilof Islands, Alaska. Estuar Coast Shelf Sci. 1983;16: 309IN1311–310319.

[48] Hunt GL. Physics, zooplankton, and the distribution of least auklets in the Bering Sea—a review. ICES J Mar Sci J Cons. 1997;54: 600–607.

[49] Hunt GL, Russell RW, Coyle KO, Weingartner T. Comparative foraging ecology of planktivorous auklets in relation to ocean physics and prey availability. Mar Ecol Prog Ser. 1998;167 Available: http://escholarship.org/uc/item/5zr3n3r0.pdf.

[50] Brown ZW, Welcker J, Harding AMA, Walkusz W, Karnovsky NJ. Divergent diving behavior during short and long trips of a bimodal forager, the little auk *Alle alle*. J Avian Biol. 2012;43: 215–226. 10.1111/j.1600-048X.2012.05484.x

[51] Pedersen CE, Falk K. Chick diet of dovekies *Alle alle* in Northwest Greenland. Polar Biol. 2001;24: 53–58. 10.1007/s003000000173

[52] Karnovsky NJ, Hobson KA, Iverson S, Hunt GL. Seasonal changes in diets of seabirds in the North Water Polynya: a multiple-indicator approach. Mar Ecol-Prog Ser-. 2008;357: 291.

[53] Fort J, Cherel Y, Harding A, Welcker J, Jakubas D, Steen H, et al Geographic and seasonal variability in the isotopic niche of little auks. Mar Ecol Prog Ser. 2010;414: 293–302.

[54] Tamelander T, Renaud PE, Hop H, Carroll ML, AMBROSE WG, Hobson KA. Trophic relationships and pelagic-benthic coupling during summer in the Barents Sea Marginal Ice Zone, revealed by stable carbon and nitrogen isotope measurements. Mar Ecol Prog Ser. 2006;310: 33–46.

[55] Søreide JE, Falk-Petersen S, Hegseth EN, Hop H, Carroll ML, Hobson KA, et al Seasonal feeding strategies of Calanus spp. in the high-Arctic Svalbard region. Deep Sea Res Part II Top Stud Oceanogr. 2008;55: 2225–2244.

[56] Gaston AJ, Jones IL. The auks: alcidae. Oxford University Press, USA; 1998.

[57] Jakubas D, Wojczulanis-Jakubas K, Iliszko L, Darecki M, Stempniewicz L. Foraging strategy of the little auk *Alle alle* throughout breeding season—switch from unimodal to bimodal pattern. J Avian Biol. 2014;45: 551–560. 10.1111/jav.00303

[58] Kwasniewski S, Gluchowska M, Jakubas D, Wojczulanis-Jakubas K, Walkusz W, Karnovsky N, et al The impact of different hydrographic conditions and zooplankton communities on provisioning Little Auks along the West coast of Spitsbergen. Prog Oceanogr. 2010;87: 72–82.

[59] Welcker J, Harding AMA, Karnovsky NJ, Steen H, Strøm H, Gabrielsen GW. Flexibility in the bimodal foraging strategy of a high Arctic alcid, the little auk *Alle alle*. J Avian Biol. 2009;40: 388–399. 10.1111/j.1600-048X.2008.04620.x

[60] Jakubas D, Iliszko LM, Strøm H, Darecki M, Jerstad K, Stempniewicz L. Foraging behavior of a high-Arctic zooplanktivorous alcid, the little auk, at the southern edge of its breeding range. J Exp Mar Biol Ecol. 2016;475: 89–99. 10.1016/j.jembe.2015.11.010

[61] Polyak L, Alley RB, Andrews JT, Brigham-Grette J, Cronin TM, Darby DA, et al History of sea ice in the Arctic. Quat Sci Rev. 2010;29: 1757–1778.

[62] Lovvorn JR, Grebmeier JM, Cooper LW, Bump JK, Richman SE. Modeling marine protected areas for threatened eiders in a climatically changing Bering Sea. Ecol Appl. 2009;19: 1596–1613. 19769106 10.1890/08-1193.1

[63] Bannasch R, Wilson RP, Culik B. Hydrodynamic aspects of design and attachment of a back-mounted device in Penguins. J Exp Biol. 1994;194: 83–96. 9317385 10.1242/jeb.194.1.83

[64] Kidawa D, Jakubas D, Wojczulanis-Jakubas K, Iliszko L, Stempniewicz L. The effects of loggers on the foraging effort and chick-rearing ability of parent little auks. Polar Biol. 2012;35: 909–917.

[65] Phillips RA, Xavier JC, Croxall JP, Burger AE. Effects of satellite transmitters on albatrosses and petrels. The Auk. 2003;120: 1082–1090.

[66] Prop J, Aars J, Bårdsen B-J, Hanssen SA, Bech C, Bourgeon S, et al Climate change and the increasing impact of polar bears on bird populations. Front. Ecol. Evol. 2015;3: 33 10.3389/fevo.2015.00033

[67] Stempniewicz L. The polar bear *Ursus maritimus* feeding in a seabird colony in Frans Josef Land. Polar Res. 1993;12: 33–36.

[68] Descamps S. Winter temperature affects the prevalence of ticks in an arctic seabird. PLoS ONE. 2013;8: e65374 10.1371/journal.pone.0065374 23750259 PMC3672161

[69] Van Hemert C, Pearce JM, Handel CM. Wildlife health in a rapidly changing North: focus on avian disease. Front Ecol Environ. 2014;12: 548–556. 10.1890/130291 PMC7164092 32313510

[70] Hovinen JEH, Welcker J, Descamps S, Strøm H, Jerstad K, Berge J, et al Climate warming decreases the survival of the little auk (*Alle alle*), a high Arctic avian predator. Ecol Evol. 2014;4: 3127–3138. 10.1002/ece3.1160 25247069 PMC4161185

[71] Beaugrand G, Luczak C, Edwards M. Rapid biogeographical plankton shifts in the North Atlantic Ocean. Glob Change Biol. 2009;15: 1790–1803. 10.1111/j.1365-2486.2009.01848.x

[72] Daufresne M, Lengfellner K, Sommer U. Global warming benefits the small in aquatic ecosystems. Proc Natl Acad Sci. 2009;106: 12788–12793. 10.1073/pnas.0902080106 19620720 PMC2722360

[73] Garzke J, Ismar SMH, Sommer U. Climate change affects low trophic level marine consumers: warming decreases copepod size and abundance. Oecologia. 2014;177: 849–860. 10.1007/s00442-014-3130-4 25413864

[74] Falkowski PG, Oliver MJ. Mix and match: how climate selects phytoplankton. Nat Rev Microbiol. 2007;5: 813–819. 17853908 10.1038/nrmicro1751

[75] Fort J, Beaugrand G, Grémillet D, Phillips RA. Biologging, Remotely-Sensed Oceanography and the Continuous Plankton Recorder Reveal the Environmental Determinants of a Seabird Wintering Hotspot. PLoS ONE. 2012;7: e41194 10.1371/journal.pone.0041194 22815967 PMC3399871

[76] Jakubas D, Wojczulanis-Jakubas K, Walkusz W. Response of Dovekie to Changes in Food Availability. Waterbirds. 2007;30: 421–428. 10.1675/1524-4695(2007)030[0421:RODTCI]2.0.CO;2

[77] Grémillet D, Fort J, Amélineau F, Zakharova E, Le Bot T, Sala E, et al Arctic warming: nonlinear impacts of sea-ice and glacier melt on seabird foraging. Glob Change Biol. 2015;21: 1116–1123. 10.1111/gcb.12811 25639886

[78] Huettmann F, Artukhin Y, Gilg O, Humphries G. Predictions of 27 Arctic pelagic seabird distributions using public environmental variables, assessed with colony data: a first digital IPY and GBIF open access synthesis platform. Mar Biodivers. 2011;41: 141–179.

