# Supplementary material for: Where to Forage in the Absence of Sea Ice? Bathymetry As a Key Factor for an Arctic Seabird
Source: PLoS One. 2016 Jul 20;11(7):e0157764. doi: 10.1371/journal.pone.0157764 (PMC4954664; doi:10.1371/journal.pone.0157764)
Supplement: S1 Fig — Red dots correspond to foraging or resting (speed <10 km.h-1) and black dots to travelling (instant speed >10 km.h-1). Sea ice extent data were downloaded from the U.S. National Ice Center (http://www.natice.noaa.gov/products/daily_products.html). White: pack ice with an ice concentration >80%. Light blue: marginal ice zone (MIZ) with an ice concentration <80%. In the marginal ice zone, sea ice concentration decreased between pack ice and open water. Only 1 out of 4 trips were complete, thus we did not include these tracks in our analyses. For the complete track, the maximum distance to the colony was 84.3 km and the trip duration was 22.4h. Projection: GR96/ UTM zone 27N. (PDF) [file pone.0157764.s001.pdf]

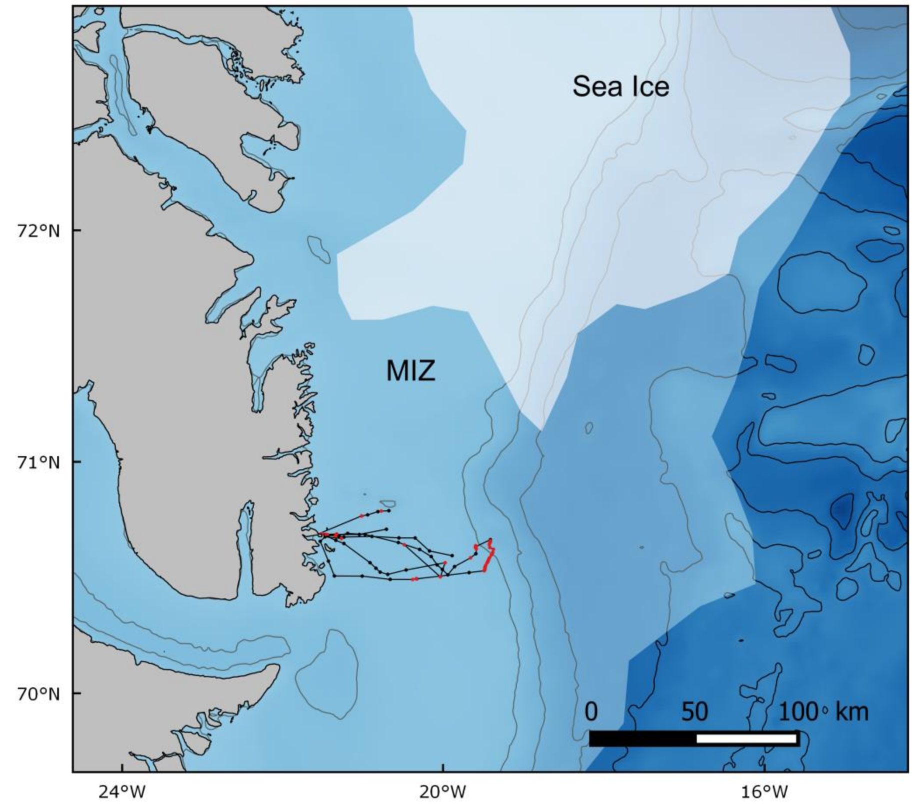

S1 Fig. GPS tracks of 3 little auks in 2011 and sea ice extent. Red dots correspond to foraging or resting (speed <10 km.h^{-1}) and black dots to travelling (instant speed >10 km.h^{-1}). Sea ice extent data were downloaded from the U.S. National Ice Center (http://www.natice.noaa.gov/products/daily_products.html). White: pack ice with an ice concentration >80%. Light blue: marginal ice zone (MIZ) with an ice concentration <80%. In the marginal ice zone, sea ice concentration decreased between pack ice and open water. Only 1 out of 4 trips were complete, thus we did not include these tracks in our analyses. For the complete track, the maximum distance to the colony was 84.3 km and the trip duration was 22.4h. Projection: GR96/ UTM zone 27N.
