# Supplementary material for: Where to Forage in the Absence of Sea Ice? Bathymetry As a Key Factor for an Arctic Seabird
Source: PLoS One. 2016 Jul 20;11(7):e0157764. doi: 10.1371/journal.pone.0157764 (PMC4954664; doi:10.1371/journal.pone.0157764)
Supplement: S2 Fig — Chicks were weighed every second day. Chick growth was compared between years during the linear growth period (delimited by dotted lines) using a linear mixed effect model with mass as an explanatory variable, chick age and year as fixed factors and chick as a random factor. Model selection process retained model with chick age as fixed factor and chick as random effect. Year factor was rejected meaning that there is no difference of chick growth between years. (PDF) [file pone.0157764.s002.pdf]

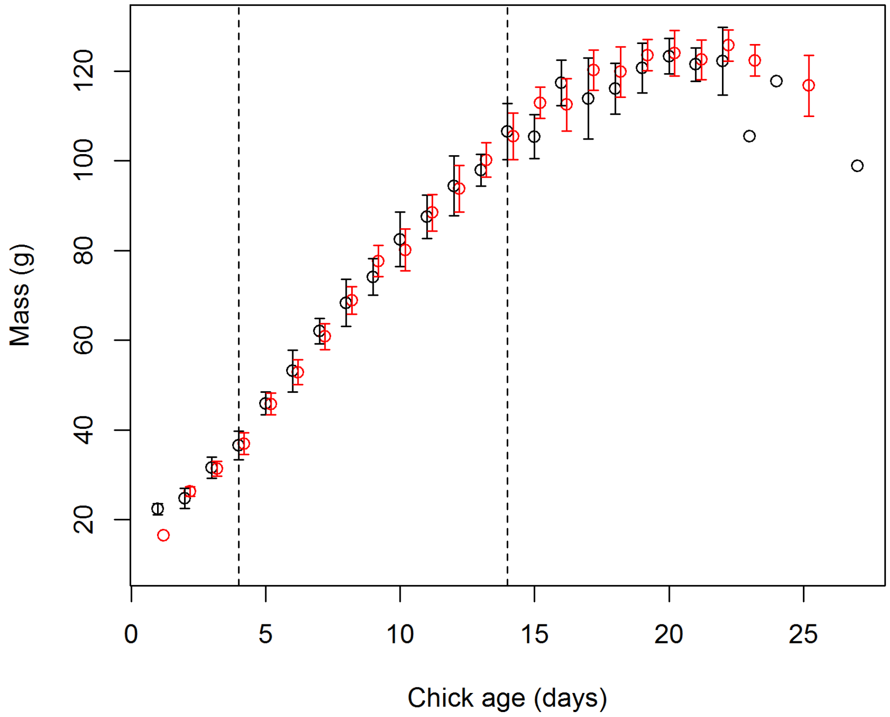

S2 Fig. Chick Growth curves for 2012 (black, n=24 chicks) and 2014 (red, n=29 chicks). Chicks were weighed every second day. Chick growth was compared between years during the linear growth period (delimited by dotted lines) using a linear mixed effect model with mass as an explanatory variable, chick age and year as fixed factors and chick as a random factor. Model selection process retained model with chick age as fixed factor and chick as random effect. Year factor was rejected meaning that there is no difference of chick growth between years.
