# Supplementary material for: Where to Forage in the Absence of Sea Ice? Bathymetry As a Key Factor for an Arctic Seabird
Source: PLoS One. 2016 Jul 20;11(7):e0157764. doi: 10.1371/journal.pone.0157764 (PMC4954664; doi:10.1371/journal.pone.0157764)
Supplement: S3 Fig — There was no difference in body condition between both years (F1,183 = 0.064, p = 0.8). (PDF) [file pone.0157764.s003.pdf]

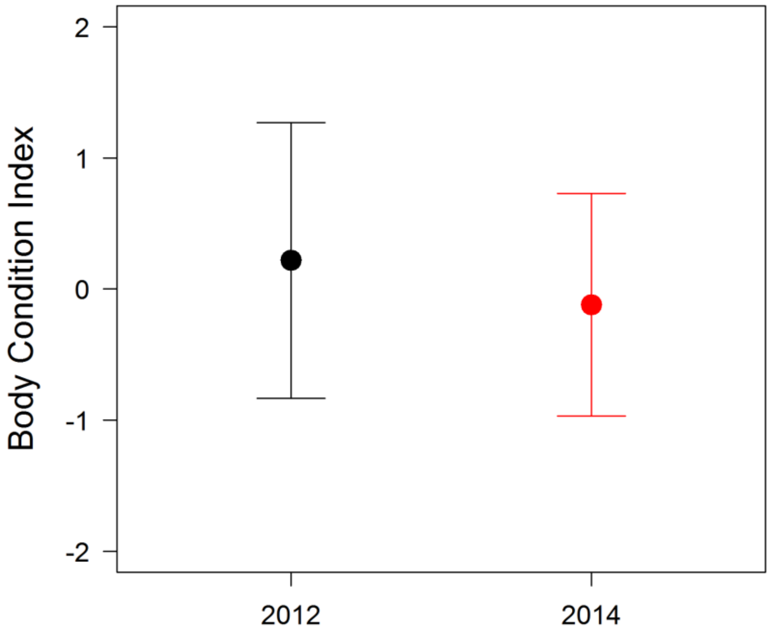

S3 Fig. Adult body condition index (mean±SE) in 2012 (black, n=65) and 2014 (red, n=120) calculated following [42]. There was no difference in body condition between both years ($F_{1,183} = 0.064$, $p=0.8$).
