# Supplementary material for: Where to Forage in the Absence of Sea Ice? Bathymetry As a Key Factor for an Arctic Seabird
Source: PLoS One. 2016 Jul 20;11(7):e0157764. doi: 10.1371/journal.pone.0157764 (PMC4954664; doi:10.1371/journal.pone.0157764)
Supplement: S1 Table — Model selection of all combination of factors, a chick random effect is included. K: number of parameters. ΔAIC is the difference of AIC between a given model and the model with the lowest AIC. Best model is number 1 with the smallest AIC and less parameters than model 2. (PDF) [file pone.0157764.s004.pdf]

S1 Table. Model selection using Akaike's information criterion (AIC) to test the effects of age and year on chick body mass (51 chicks, 256 observations)

N°	Model	K	AIC	ΔAIC
1	Age	4	1653.0	0.0
2	Age+Year	5	1655.0	2.0
3	Year	4	2344.2	691.3
4	Intercept	3	2342.5	689.5

Model selection of all combination of factors, a chick random effect is included. K: number of parameters. ΔAIC is the difference of AIC between a given model and the model with the lowest AIC. Best model is number 1 with the smallest AIC and less parameters than model 2.
