# Supplementary material for: Where to Forage in the Absence of Sea Ice? Bathymetry As a Key Factor for an Arctic Seabird
Source: PLoS One. 2016 Jul 20;11(7):e0157764. doi: 10.1371/journal.pone.0157764 (PMC4954664; doi:10.1371/journal.pone.0157764)
Supplement: S2 Table — (PDF) [file pone.0157764.s005.pdf]

S2 Table. Parameter estimation of model 1 testing the effects of age on chick body mass.

Parameter	Estimate	SE
Intercept	12.6	1.3
Chick age	6.8	0.1
